# Impact of Heat Stress on the Balance between Oxidative Markers and the Antioxidant Defence System in the Plasma of Mid-Lactating Modicana Dairy Cows

**DOI:** 10.3390/ani14142034

**Published:** 2024-07-10

**Authors:** Daniela Alberghina, Annalisa Amato, Giacoma Brancato, Carmelo Cavallo, Luigi Liotta, Vincenzo Lopreiato

**Affiliations:** Department of Veterinary Sciences, Università degli Studi di Messina, Viale Palatucci 13, 98168 Messina, Italy; annalisa.amato@studenti.unime.it (A.A.); carmelo.cavallo@studenti.unime.it (C.C.); luigi.liotta@unime.it (L.L.); vincenzo.lopreiato@unime.it (V.L.)

**Keywords:** THI, milk, reactive oxygen metabolites, antioxidant vitamins, plasma metabolites, dairy cows

## Abstract

**Simple Summary:**

This study was conducted to determine the effects of the incremental temperature humidity index (THI) on oxidant and antioxidant plasma markers in mid-lactating dairy cows. Results showed a significant increase in oxidative markers and a significant decrease in antioxidant defence; this balance results in oxidative stress. Heat stress significantly modulated fat milk content, blood cell number, and plasma metabolite concentration.

**Abstract:**

Animal health is affected during heat stress as a result of impaired immune responses, increased production of reactive oxygen species, and/or a deficiency of antioxidants. This leads to an imbalance between oxidants and antioxidants and results in oxidative stress. Heat stress is usually measured in dairy cattle via the temperature-humidity index (THI). In the present study, we aimed at assessing the influence of incremental THI on the balance between oxidative markers and the antioxidant defence system in the plasma of Modicana cows. Twenty-four multiparous, mid-lactating dairy cows were divided into two groups on the basis of different levels of mean THI reached in the period of the previous week up until the day of blood and milk sampling (April THI_1_:55, May THI_2_:68, June THI_3_:71, July THI_4_:80). The blood samples were collected to measure reactive oxygen metabolites (ROM) and advanced oxidation protein products (AOPP) on the one hand, and antioxidant defense markers (ferric reducing ability of plasma (FRAP), paraoxonase (PON), plasma thiol groups (SHp)), as well as lipid-soluble antioxidant pro-vitamin (β-carotene) and vitamins (tocopherol and retinol) on the other hand. Milk characteristics, haematological values, and plasma biochemical metabolites were also evaluated. Results showed a significant increase in ROM (*p* < 0.05) and a significant decrease in PON (*p* < 0.05), AOPP (*p* < 0.05), and β-carotene (*p* < 0.001). Incremental THI significantly decreased levels of milk fat content, red and white blood cells, plasma glucose, and non-esterified fatty acids, while significantly increasing monocytes and the concentrations of β-hydroxybutyrate and creatinine, but not fructosamine. The results of the study show that heat stress significantly affects reactive oxygen species production and antioxidant parameters. Carotenoid supplementation should be considered to alleviate the impact of these effects.

## 1. Introduction

Livestock health and productivity are particularly susceptible to the negative effects of rising temperatures, especially in cases of drought. Although nowadays science is studying to adopt several strategies to improve the environmental condition and the resilience of the animals with the use of management strategies (e.g., sprinklers, fans), nutritional strategies, and innovative systems of watering, it is well demonstrated how genetics plays a fundamental role. Heat stress reduces dry matter intake, negatively affecting milk yield and composition, nutrient absorption, and, as a consequence, impacting energy metabolism, the immune system, and the inflammatory response [1]. In the near future, animal life will be coping with heat waves that have increased intensity and last for longer than even the most devastating heat wave experienced to date [2]. Jacobs et al. used a heat wave simulator, where mice were first maintained at control temperatures for five days, then transitioned to temperatures that oscillated between a minimum of 22 °C and a maximum of 34 °C for one day, followed by heat wave temperatures which oscillated between a minimum of 24 °C and a maximum of 39 °C for a period of three full days. Heat wave exposure caused oxidative stress in different organs, which may have had negative consequences for animal physiology and life expectancy [3]. Similar temperature conditions to those in the previous study occurred naturally in Sicily; in particular, the ambient temperature reached 50 °C in the second half of July 2023 [4]. Dairy cattle are more susceptible to heat stress compared to other livestock species. This susceptibility is due to breeding selections aimed at boosting milk production, which in turn increases metabolic heat output [5]. Dairy cows with high levels of productivity, begin to lose the ability to regulate their body temperature when ambient temperatures surpass 30 °C [6]. Heat stress is usually measured in dairy cattle via the temperature-humidity index (THI). THI presents the combination of ambient temperature and relative humidity and is a useful and easy way to assess the risk of heat stress [7]. A THI below 70 is generally considered to be comfortable, between 75 and 78 stressful, and above 78 hazardous [5]. Above this threshold, cattle try to maintain body temperature by dissipating heat and undertaking numerous behavioural and physiological changes. These changes negatively impact the production, reproduction, and health of animals, in turn leading to economic losses for the farm. Specifically, the cow’s immune system is strictly compromised since heat stress decreases the activity and the number of several neutrophils and lymphocytes [8]. Furthermore, high temperatures can induce oxidative stress, a condition resulting from an imbalance between the production of reactive oxygen species (ROS) and antioxidants. Normally, antioxidants can neutralise excessive ROS to maintain redox balance [9]. Heat-induced disruption of this balance leads to an increase in reactive oxygen metabolites (ROM), which damage cells and tissues and impair immune function [10,11]. Unlike ROS, ROM is more stable and can be easily quantified [12]. Additionally, the compromise of the immune system may result in an increased susceptibility of cows to several diseases. Along with ROM, various blood biomarkers are commonly measured and studied to assess the condition of oxidative stress in cows.

FRAP (Ferric Reducing Ability of Plasma) measures the capacity of plasma to reduce Fe^3+^ (ferric ion) to Fe^2+^ (ferrous ion) based on the presence of available reducing agents. FRAP offers a putative index of the antioxidant, or reductive, potential of plasma during oxidative stress [13].

Paraoxonase (PON) is an antioxidant enzyme whose activity is decreased in certain conditions associated with oxidative stress [14]. New compounds that can serve as biomarkers have been identified. Among these are advanced oxidation protein products (AOPPs), which are primarily formed by chlorinated oxidants produced through the activity of myeloperoxidase [15]. However, these AOPPs have not yet been evaluated during thermal stress in dairy cows. Specific protein cysteine thiols (SHp) have emerged as markers of redox status levels [16]. Evaluating the levels of SHp could be interesting in understanding the response to thermal stress in dairy cows.

Carotenoids are a fascinating group of natural pigments. Not only are they responsible for a broad array of coloration in nature, but, more importantly, they have key functional roles in biology [17]. They are precursors of vitamin A and present a robust antioxidant capacity that contributes to protecting the body against the effects of ROS [18]. Similar to, carotenoids, vitamin E is an essential antioxidant [19].

A better understanding of the physiological variation in lactating dairy cows during extreme environmental conditions can help to improve their welfare and prevent thermal stress-related economic loss. The immune system is the body’s primary defence mechanism for protecting against and coping with environmental stressors. White blood cells (WBCs), red blood cells (RBCs), haemoglobin (Hb), packed cell volume (PCV), glucose, and protein concentration in the blood are usually affected by thermal stress [20]. Another potential biomarker of stress is fructosamine, a glycated protein, as it reflects long-term blood glucose concentration [21]. 

Within Italian autochthonous breeds, the Modicana is the most important native bovine breed in Sicily, both in terms of consistency and zootechnical quality. In particular, Modicana’s milk is used to produce a cheese with a protected designation of origin (PDO) label, named “Ragusano”, which is aged from 4 to 12 months. Modicana cows are usually reared in extensive systems, using pasture during the grazing season and with limited or no supplementation of concentrate to their diet. Semi-intensive farming practices are also possible when animals cannot go to pasture and need higher concentrate supplementation [22]. Modicana cows are appreciated by breeders for their maternal behaviours and for their adaptability to adverse conditions, dietary in particular, and their milk has higher levels of biomolecules and antioxidant activity compared to Holstein [23]. Due to the rusticity of this breed and the hypothesis of its greater resilience to adverse weather conditions, the objective of this study was to investigate the effects of thermal stress on the balance between oxidative markers and antioxidant defence in the plasma of Modicana cows.

## 2. Materials and Methods

### 2.1. Animal Management and Treatment

The present study was performed in a commercial dairy farm with the Modicana breed located in the province of Ragusa, Sicily, Italy (36°56′49″ N 14°41′50″ E, 500 mt above sea level) under the traditional semi-intensive farming practice. His dairy cattle farm was monitored from April to July 2022. The study was approved by the Ethics Committee of the Department of Veterinary Sciences, University of Messina, code number 041/2020. A total of 24 healthy lactating Modicana dairy cows [Body Condition Score (BCS): 2.75 ± 0.15; parity: 3.17 ± 1.49; days in milk (DIM): 158 ± 37 d; and milk yield (MY): 11.38 ± 3.7 kg/d (mean ± SD)] were selected: two groups each for evaluation on two different environmental conditions of THI (Group 1 April, May, Group 2 June, and July). The cows in this study were examined daily for health-related problems via visual observation, a temperature check, and monitoring milk yield by trained personnel (see Table 1 for individual characteristics). Cows were fed hay ad libitum and concentrate according to their milk production (on average 8.7 kg/head/day as dry matter of concentrate in two equal meals in the morning and afternoon during the milking). Feed formula and nutrients are reported in Table 2. The cows were milked twice daily (6 a.m. and 6 p.m.). After each milking, cows were allowed to pasture for the entire period under investigation (for a minimum of 6 h during daylight, from 8:00 a.m. to 2:00 p.m.). The animals had free access to water, both indoors and outdoors (the indoor housing was a free-stall barn). The animals were grouped based on body condition score (BCS) and parity and then assigned to two groups to ensure similar days in milk and milk yields (n = 12 animals per group). The selected animals were multiparous, mildly lactating dairy cows and were evaluated for blood and milk parameters across four periods.

### 2.2. Environmental Data

Climate data used for this study was obtained on an hourly basis from a weather station installed in Ragusa (36°56′ N, 14°44′ E). The mean temperature (T) and relative humidity (RH) recorded from the previous week until the day of sampling (T_1_ = 11 April, T_2_ = 27 May, T_3_ = 17 June, and T_4_ = 26 July) were used to calculate the related temperature humidity index (THI) using a formula previously described [24]:

THI = (1.8 × T + 32) − [(0.55 − 0.0055 × RH) × (1.8 × T − 26.8)]

where T is the air temperature (°C) and RH is the relative humidity (%).

### 2.3. Milk Performance and Analysis

Consecutive evening and morning milk samples were individually collected monthly (April, May, June, and July) with a manual milk sampler (Waikato Milk Metre, Milkline Company, Piacenza) and composited in proportion to milk yield. Milk yield was recorded, and individual milk samples (60 mL) were collected to assess milk quality (fat, protein, casein, and lactose) using a Fourier transform infrared analyzer (Milkoscan FT2, FOSS, Hillerød, Denmark).

### 2.4. Blood Sample Collection and Analysis

For each cow, blood samples were collected monthly before the morning feed from the jugular vein using an 18-gauge Vacutest Kima needle (Vacutest Kima SRL, Arzergrande, Italy). The samples were drawn into two types of tubes: 9-mL evacuated test tubes containing lithium heparin and 6-mL K-EDTA tubes (Vacumed, Padova, Italy). The collected samples were immediately cooled in an ice-water bath. After collection, the blood samples were collected into lithium heparin tubes centrifuged at 1900× *g* for 16 min at 4 °C. The resultant plasma was aliquoted and stored at −20 °C until further analysis. Plasma metabolites were analysed at 37 °C by using an automated clinical analyzer (ILAB 650, Instrumentation Laboratory Company, Lexington, MA, USA), as reported by Lopreiato et al. [25]. Commercial kits were used to measure glucose, total cholesterol, urea, calcium, inorganic phosphorus, total protein, albumin, total bilirubin, and creatinine (Instrumentation Laboratory SpA, Milan, Italy), nonesterified (free) fatty acids (FFA), and β-hydroxybutyrate (BHB) (kit Ranbut, Randox Laboratories Ltd., Crumlin, UK). The total globulin fraction was determined by subtracting albumin from the total protein. The ratio of albumin to globulin A/G was then calculated. ROM was measured as described by Bionaz et al. [26]. Plasma paraoxonase (PON, EC 3.1.8.1) activity was assessed by adapting the method of Ferre et al. [27] to the ILAB 650. Ferric-reducing antioxidant power (FRAP) was measured using the colorimetric method of Benzie and Strain [13] and advanced oxidation protein products (AOPP) as described by Hanasand et al. [28]. Plasma thiol groups (SHp) were determined by titration with 5,5-dithiobis-2-nitrobenzoic acid using a commercial kit (Diacron, Italy). Plasma retinol, tocopherol, and β-carotene were extracted with hexane and analysed by reversed-phase HPLC using Spherisorb ODS-2, 3 μm, in a 150 × 4.6 mm column (Alltech, Deerfield, IL, USA); a UV detector set at 325 nm (for retinol), 290 nm (for tocopherol), or 460 nm (for β-carotene); and 80:20 methanol: tetrahydrofuran as the mobile phase. 

K-EDTA tubes were processed using a clinical autoanalyzer. Samples were analysed within 2 h of collection using an ADVIA 2120 Haematology System machine (Siemens, Germany). The blood parameters analysed were the following: red blood cell count (RBC), hematocrit value (HCT), haemoglobin concentration (HGB), platelet count (PLT), total white blood cell (WBC) count, WBC differential count for neutrophils, eosinophils, basophils, lymphocytes, and monocytes as an absolute number.

The data supporting the findings of this study are available as Supplementary Materials file.

### 2.5. Statistical Analysis

Significance analysis of the environmental parameters, milk yield, milk composition, biomarkers of oxidative stress, blood cells, and plasma metabolites at different time points was conducted using one-way ANOVA supplemented by Dunnett’s multiple comparisons post-hoc test compared to T4, with analysis performed using GraphPad Prism v8.4.2 (GraphPad Software, Inc., La Jolla, CA, USA). Results are presented as the mean ± standard deviation (SD). A statistically significant difference was defined as *p* < 0.05 and highly significant at *p* < 0.01.

## 3. Results

### 3.1. Environmental Conditions

**Figure 1:**
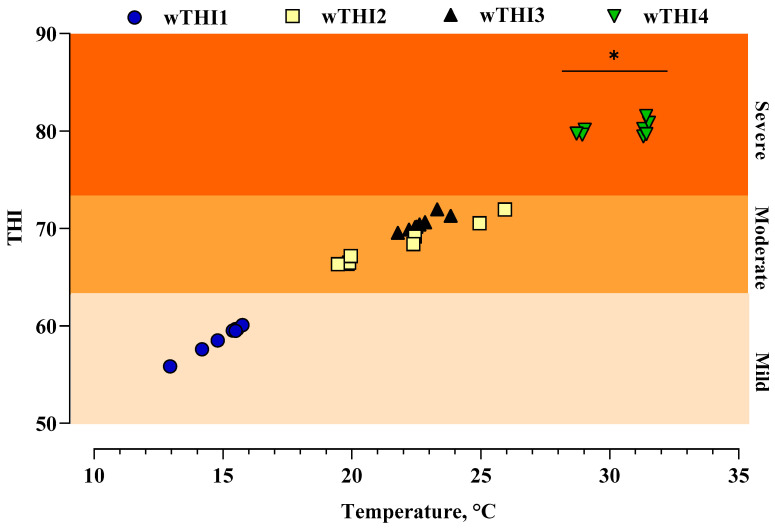
Mean daily values of weekly temperature (°C) and weekly temperature-humidity index (wTHI) during the experimental period are presented. Asterisk indicate statistical difference compared to previous periods (* *p* < 0.001).

Mean values of daily ambient temperature, relative humidity, and THI measured during the weeks of time 1, time 2, time 3, and time 4 in the period of April–July 2022 are presented in Table 3. The average daily temperature (30.45 ± 1.29 °C) and THI (80.15 ± 0.70) were significantly higher (*p* < 0.001) in T_4_ than T_1_, T_2_, and T_3_ (Figure 1).

### 3.2. Milk Parameters

As shown in Table 4, although no differences were detected between groups for milk yield, protein, and lactose concentrations, the milk fat concentration was significantly decreased in the THI_3_ group (*p* < 0.05), whereas the highest value was observed at THI_1_, when Modicana cows were in a condition of thermal comfort (THI = 55).

### 3.3. Haematology, Biomarkers of Energy, Muscle Body Mass, and Liver Function

As shown in Table 5, there was a significant decrease in both RBC and WBC counts (*p* < 0.05). The RBC count was significantly lower during wTHI4 compared to wTHI1 and wTHI2 (*p* < 0.05 and *p* < 0.01, respectively). WBC count, particularly eosinophils, was significantly higher during wTHI2 compared to wTHI4 (*p* < 0.05). The number of monocytes significantly increased (*p* < 0.01), with significant differences observed at wTHI4 compared to wTHI1 (*p* < 0.05) and wTHI2 (*p* < 0.01). Plasma glucose and NEFA decreased significantly (*p* < 0.001 and *p* < 0.01, respectively), while BHB and creatinine significantly increased (*p* < 0.05 and *p* < 0.001, respectively) (see Table 6). The highest glucose values were observed in wTHI1 and the lowest in wTHI4 (*p* < 0.01), while the lowest BHB values were in wTHI1 (*p* < 0.05) and the highest creatinine values were in wTHI4 (*p* < 0.01).

### 3.4. Oxidative Biomarkers and Antioxidant Parameters in Plasma

**Figure 2:**
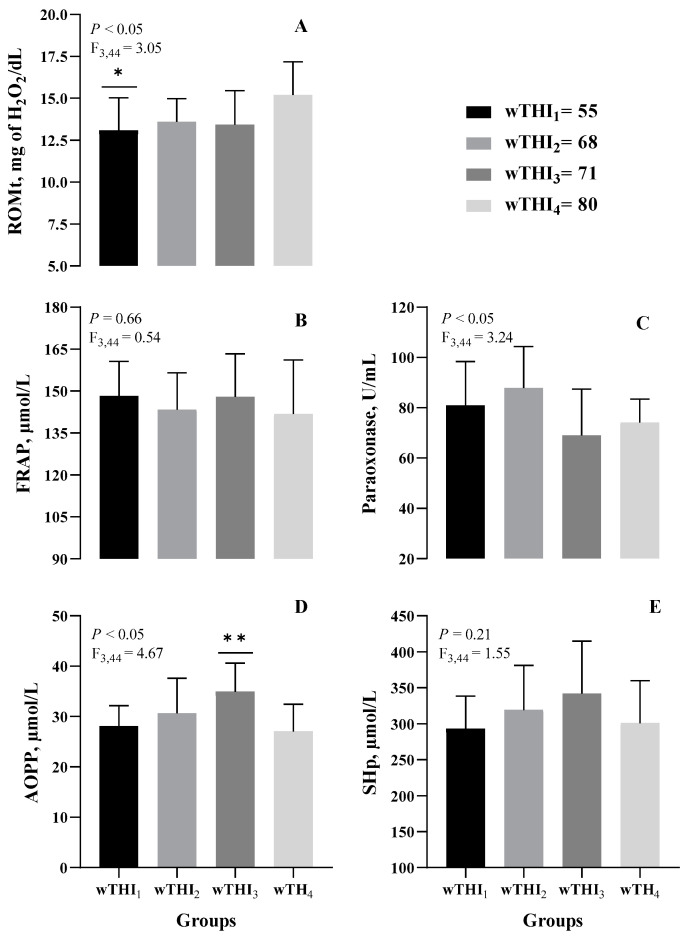
Effect of weekly temperature-humidity index (wTHI) on plasma biomarkers related to oxidative stress and antioxidant defence in Modicana mild lactating cows (**A**) reactive oxygen metabolite totals (ROMt), (**B**) ferric reducing ability of plasma (FRAP), (**C**) paraoxonase (PON), (**D**) advanced oxidation protein products (AOPP), (**E**) plasma thiol groups (SHp). Asterisks indicate statistical differences vs. THI_4_ (* *p* < 0.05; ** *p* < 0.01).

**Figure 3:**
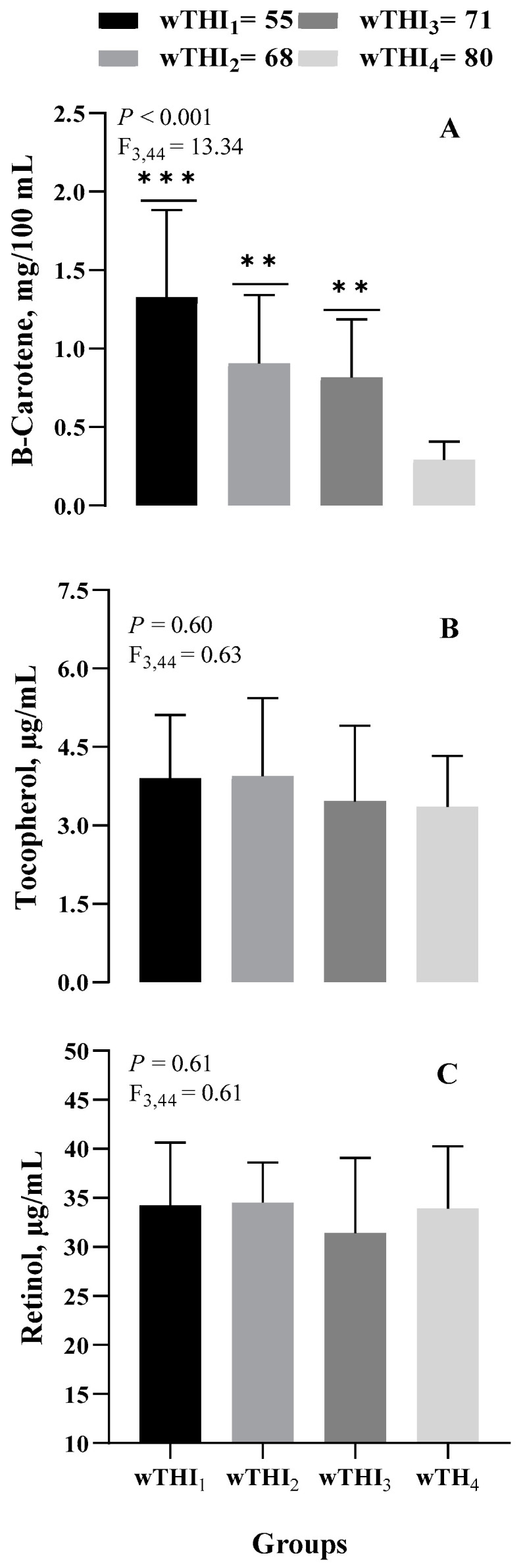
Effect of weekly temperature-humidity index (wTHI) on lipid-soluble antioxidant pro-vitamin ((**A**) β-carotene) and vitamins ((**B**) tocopherol, (**C**) retinol) in the plasma of Modicana mild lactating cows. Asterisks indicate statistical differences vs. wTHI4 (** *p* < 0.01; *** *p* < 0.005).

As shown in Figure 2, significant changes were observed in the levels of ROM, which significantly increased (*p* < 0.05), and in the activity of PON and AOPP levels, which significantly decreased (*p* < 0.05) as an effect of incremental wTHI. A significant and progressive decrease of carotenoids was found during the experimental period (*p* < 0.001; Figure 3). Dunnett’s multiple comparison post-hoc test revealed significant differences for each group compared to wTHI_4_.

## 4. Discussion

This study aimed to evaluate the effects of the incremental temperature-humidity index (THI) on haematological values, biochemical metabolites, and the balance between oxidative markers and the antioxidant defence system in the plasma of Modicana dairy cows. A common belief among breeders of the Modicana breed is that cows are not affected by heat stress because it is a rustic breed. Confirming these beliefs, Modicana cows showed no differences in milk yield from April to July. This is an interesting result, since several previous studies demonstrated how heat stress decreases the production of milk [29,30]. In fact, it is well known that high yielding cows are more challenged by heat stress than lower yielding cows [31], since the genetic selection for high milk production reduces their thermoregulatory ability [32]. On the other hand, Modicana cows, being less productive, can better cope with heat stress (producing less metabolic heat). Numerous studies had previously demonstrated the changes in milk composition in dairy cows under heat stress [33,34,35].

In the current study, milk protein and lactose concentration were not affected by different levels of THI, and this was in accordance with previous studies in buffalo and cow milk [36,37]. However, the milk fat content decreased when THI increased, as consistent with previous studies [37,38,39].

This could be related to a decrease in dry matter intake in cows during the hottest period, which resulted in a decreased intake of fibre [40] probably as a consequence of reduced acetate production in the rumen. Unfortunately, we have no data on dry matter intake.

The RBC count decreases in T_4_, and this change could be an effect of the destruction of erythrocytes by oxidative stress [41]. If a slight upward trend in bilirubin levels were to have been found, this explanation could be plausible. The decrease of RBC but not of PCV could indicate that a slight dehydration, masked by the concomitant presence of anaemia, occurred in these animals. The concomitant but not significant increase in total proteins, without alteration of the A/G ratio, also confirms this hypothesis. Although A/G is within the range, it shows lower values than those previously reported [42]. The fact that there is fibrinogen in the plasma compared to the serum could explain this result. Heat stress has been proven to decrease the activity and quantity of specific immune cells, such as neutrophils and lymphocytes, making cows more susceptible to infections and illness [11]. Eosinophils respond differently to heat stress, and it has been documented that there is a reduction in the eosinophil counts in sheep exposed to high temperatures [43,44]. A significant increase in monocytes has been found. Monocyte numbers are variable in cattle and are thus not a sensible indicator for a specific disease, but usually monocytosis has been observed during acute stress [45]. Monocytes play a pivotal role in defence against infection or injury, and they are responsive to heat stress in humans [46].

The increased plasma creatinine concentrations observed in this and earlier studies [39,47] might be primarily due to a reduction in renal clearance rather than increased muscle catabolism, as urea levels remained unchanged. However, heat stress slightly increased levels of fructosamine, but in our study, it failed to be a marker of stress. Recently, elevated fructosamine levels have been reported in dairy cows under chronic stress [21].

Increased BHBA blood levels, as well as decreased glucose levels, suggest a negative energy balance [48]. Energy balance is the difference between energy consumed and energy used for both maintenance and production. When energy expenditure exceeds intake, this results in a state of Negative Energy Balance (NEB) [49]. For heat stress metabolism during mid-lactation, there is no increase in NEFA concentrations; this finding agrees with some previous studies [50,51]. Oxidative metabolism of fatty acids is essential for creating energy for various biological functions, but it also ROM, or free radicals, that can be destructive to cells and tissues [52]. When ROMs are produced faster than they can be neutralised by antioxidant mechanisms, oxidative stress can result. There are increasing numbers of reports that suggest the involvement of ROM in a variety of pathologic and physiological events. A tendency for an increase in ROM levels has been found in sheep as an effect of thermal stress [53]. In transition cows, those with a high body condition score showed neither a change in plasma ROM levels [54] nor higher levels [55]. In Holstein cows exposed to a nutritional and environmental stress challenge, the variation in ROM was significant [56]. To the best of the author’s knowledge, an increase in ROM levels related to heat stress in dairy cows, as observed in this study, has not yet been reported. Bernabucci et al. [57] found that the effect of heat stress (THI = 75) on the oxidative status in transition dairy cows by using plasma markers does not give enough information to reach definitive conclusions. The difference found in this study compared to the previous could be related to the different breed, stage of lactation, or, more likely, to the higher values of THI reached.PON is a calcium-dependent esterase that is suggested to play a protective role under oxidative stress. However, there is no information in the literature about the alteration of PON levels in thermal oxidative stress. Recent studies have reported that PON acts as an antioxidant enzyme, decreasing oxidative stress by preventing the formation of free radicals. There is an inverse relationship between lipid peroxidation and PON activity [58]. This could explain the significant reduction in PON activity found at higher THI value. In cows, a reduction in the ability of the liver to cope with increased metabolic demand near parturition can be diagnosed by a decrease in baseline plasma PON levels [26].

In dairy heifers, PON activity was significantly decreased after calving in both summer and winter groups [14]. In rats, a decrease was found after exercise with increasing temperature [59], but, to our best knowledge, this is the first report of a significant decrease in PON during thermal stress in mild lactating cows.

Higher concentrations of AOPP in T_3_ suggest that these animals suffered oxidative stress and damage to proteins when exposed to heat stress. The observed elevation of AOPP is of particular interest, as AOPP is a marker of protein oxidation and is also considered to mediate proinflammatory responses [60]. In dairy cows, AOPP concentrations are associated with embryonic losses and are considered an indicator of acute inflammation and oxidative stress [61]. It has been observed that the concentration of AOPP increases in dairy cows when they are fed maize silage [61] and in growing dairy calves [62], possibly due to lower levels of antioxidants in silage. An increase in AOPP as a response to thermal stress in sheep has been found [53]. FRAP and SHp seem not to be clearly reflective of their involvement in the response to thermal stress in our study conditions. However, the significant decrease in carotenoids due to oxidative stress underscores the necessity of providing carotenoid supplementation during thermal stress. Vitamin E and β-carotene blood levels varied considerably between lactation stages and between countries [60]. In Holstein cows, levels of tocopherol and carotenoids are higher during mid-lactation than during other lactation stages [63]. The β-carotene levels found in this study are very low in T_4_. This is likely because carotenoid intake declined as fresh forage decreased from T1 to T4, and also because β-carotene is more heavily utilized as an antioxidant in response to the increase in NEB and ROM. A higher blood β-carotene level at the time of artificial insemination increased the pregnancy rate and reduced pregnancy loss [64]. Since a large proportion of the cows in the present study were considered deficient in β-carotene, dietary supplementation of this nutrient appears to deserve more attention in practical nutrition. Carotenoid supplementation may also help modulate the antioxidant effects of PON [65].

## 5. Conclusions

In summary, our results clearly show that the progressive increase in THI causes an imbalance between oxidative markers and the antioxidant defence system in Modicana cows, leading to a decrease in milk fat content. The common opinion that Modicana cows are not affected by heat stress must be reconsidered in light of these results. Limitations should be acknowledged when interpreting the substantive findings of this study: body temperature measurements of the studied cows consistently remained within normal ranges, and observations of heat stress behaviours were not conducted. Nevertheless, the results obtained indicate the necessity for implementing environmental management and nutritional supplementation in dairy cow housing in Sicily to assist cows in adapting to increasingly frequent thermal stress

## Figures and Tables

**Table 1:** Names, number of lactations, and days in lactation (DIM) of Modicana dairy cows involved in the study.

Group 1	Number of Lactations	DIM at T_1_	Group 2	Number of Lactations	DIM At T_3_
Fortunata	4	147	Ardua	4	126
Gioia	3	137	Effe	3	151
Ibla	2	152	Elisea	3	150
Iblea	2	122	Elvana	3	111
Iena	2	148	Formia	5	115
Imperia	2	133	Irianna	2	144
Messina	3	84	Lisa	2	127
Angela	6	110	Luisa	2	146
Batia	5	112	Melinda	2	217
Delia	5	110	Michelle	2	146
Elisa	3	180	Nina	2	123
Zeta	4	110	Siciliana	5	108
Mean ± s.d.	3.42 ± 1.38	129 ± 26		2.91 ± 1.16	139 ± 29

**Table 2:** Ingredients and chemical composition of the diet of Modicana cows.

Ingredients, % of DM	
Corn meal	40
Roasted soybean flour	16.5
Barley meal	12
Beetpulp	9
Wheat bran	6
Sunflower meal.	6
RUMEN Bypass Fat	2.5
Minerals and Vitamins Mix	1.5
Calcium Carbonate	1.3
Saccharomyces dried yeast	1
Cane molasses	1
Na bicarbonate	1
Na chloride	0.7
P dicalcium	0.6
NutriGen 40 C	0.5
Mg oxide	0.4
**Chemical composition, % of DM**	
Crude Protein	17.7
Fat	5.01
Starch	45.64
Crude Fibre	4.40
Ash	9.84
Nel, Mcal/kg of DM	1.81

**Table 3:** Mean weekly Temperature-Humidity Index (wTHI) values and number of days with THI mean above 75 points during the week previous the blood and milk sampling day (T1, T2, T3, and T4) in Modicana mild-lactating cows (Ragusa, Italy, spring–summer 2022).

Week	Time	T °C min	T °C max	T °C mean	Hr (%) min	Hr (%) max	Hr (%) mean	wTHI min	wTHI max	wTHI mean	n. Days THImax 75–78	n. Days THImax > 78
4–11 April	T_1_	12	18	15	55	83	72	50	59	55	0	0
20–27 May	T_2_	19	25	22	43	82	64	63	74	68	1	2
10–17 June	T_3_	20	27	23	50	88	72	65	79	71	3	4
19–26 July	T_4_	27	35	30	28	83	59	71	91	80	0	7

**Table 4:** Effect of temperature-humidity index (THI) increase on milk performance of Modicana mild-lactating cows.

wTHI Periods					
Item	wTHI_1_ 55	wTHI_2_ 68	wTHI_3_ 71	wTHI_4_ 80	*p*-Values
Milk yield, kg/d	13.45 ± 3.92	11.97 ± 3.95	13.49 ± 3.42	11.33 ± 2.77	0.38
Fat, g/100 g	4.72 ± 0.81	3.64 ± 1.41	3.35 ± 1.40	3.73 ± 0.62	<0.05
Proteins, g/100 g	3.94 ± 0.33	3.84 ± 1.18	3.48 ± 1.30	3.73 ± 0.62	0.67
Caseine, g/100 g	3.01 ± 0.28	2.85 ± 0.32	2.83 ± 0.30	2.96 ± 0.19	0.32
Lactose, g/100 g	4.64 ± 0.22	4.79 ± 0.16	4.89 ± 0.34	4.82 ± 0.17	0.07

**Table 5:** Effects of the weekly value of the temperature-humidity index (wTHI mean) on haematology of Modicana mild-lactating cows.

wTHI Periods					
Items	wTHI_1_ 55	wTHI_2_ 68	wTHI_3_ 71	wTHI_4_ 80	*p*-Values
PCV, %	28.79 ± 3.86	32.02 ± 4.30	29.93 ± 7.39	28.93 ± 4.18	1.01
RBC, M/μL	6.57 ± 0.82 *	7.19 ± 0.89 **	6.21 ± 1.97	5.30 ± 0.69	<0.01
Hb, g/dL	9.33 ± 1.16	10.02 ± 1.20	9.59 ± 1.36	8.93 ± 1.14	0.18
PLT, K/μL	396 ± 91	299 ± 66	366 ± 99	360 ± 115	0.09
WBC, K/μL	10.57 ± 2.49	12.83 ± 1.57 *	10.81 ± 1.82	10.45 ± 1.66	<0.05
Neutrophils, K/μL	4.21 ± 1.42	5.60 ±1.23	4.56 ±1.66	4.54 ± 1.33	0.10
Lymphocytes, K/μL	6.06 ± 1.15	5.12 ±1.58	5.63 ±1.18	4.08 ± 1.18	0.14
Eosinophils, K/μL	0.98 ± 0.51	1.66 ±1.12 *	0.98 ± 0.80	0.76 ± 0.37	<0.05
Monocytes, K/μL	0.23 ± 0.06 *	0.27 ± 0.05	0.20 ± 0.10 **	0.34 ± 0.12	<0.01

* Dunnette multiple comparisons test differences vs. wTHI_4_ (* *p* < 0.05; ** *p* < 0.01).

**Table 6:** Effects of the weekly value of temperature-humidity index (wTHI mean) on energy, body mass, and liver function biomarkers in the plasma of Modicana mild lactating cows.

wTHI Periods					
Items	wTHI_1_ 55	wTHI_2_ 68	wTHI_3_ 71	wTHI_4_ 80	*p*-Values
Glucose, mmol/L	3.94 ± 0.23 **	3.57 ± 0.28	3.72 ± 0.28 *	3.41 ± 0.33	<0.001
Fructosamine, µMol/L	261 ± 24	277 ± 27	277 ± 20	281 ± 17	0.17
NEFA, mmol/L	0.06 ± 0.02	0.05 ± 0.01	0.09 ± 0.05	0.05 ± 0.01	<0.01
BHB, mmol/L	0.45 ± 0.14 *	0.51 ± 0.10	0.61 ± 0.20	0.62 ± 0.17	<0.05
Creatinine, mmol/L	87.19 ± 5.37 **	102 ± 7.32	105 ± 13.2	109 ± 7.83	<0.001
Urea, mmol/L	4.88 ± 0.93	4.89 ± 0.75	5.26 ± 1.08^*^	4.83 ± 0.81	0.10
Cholesterol, mmol/L	4.05 ± 0.90	4.33 ± 0.37	3.40 ± 0.92	3.75 ± 1.03	0.10
Total protein, g/L	85.8 ± 6.8	90.5 ± 7.7	92.5 ± 0.37	91.06 ± 5.52	0.08
Albumin, g/L	31.41 ± 3.14	33.26 ± 3.59	31.37 ± 2.33	31.13 ± 3.38	0.33
A/G ratio	0.59 ± 0.09	0.59 ± 0.11	0.52 ± 0.07	0.52 ± 0.07	0.10
Bilirubin, µmol/L	1.00 ± 0.35	1.10 ± 0.23	1.19 ± 0.78	1.20 ± 0.79	0.29

* Dunnette multiple comparisons test differences vs. wTHI_4_ (* *p* < 0.05; ** *p* < 0.01).

## Data Availability

Data reported in this study are available as Supplementary Materials file.

## Supplementary Materials

The following supporting information can be downloaded at: https://www.mdpi.com/article/10.3390/ani14142034/s1, Data sets.
